# Screening for depression in chronic haemodialysis patients as a part of care in dialysis setting: a cross-sectional study

**DOI:** 10.3389/fpsyt.2024.1410252

**Published:** 2024-07-23

**Authors:** Alicja Kubanek, Marcin Renke, Beata R. Godlewska, Przemysław Paul, Mateusz Przybylak, Anna S. Kowalska, Piotr Wąż, Michał Błaszczyk, Aleksandra Macul-Sanewska, Przemysław Rutkowski, Kamila Czarnacka, Krzysztof Bednarski, Jakub Grabowski

**Affiliations:** ^1^ Department of Occupational, Metabolic and Internal Diseases, Medical University of Gdańsk, Gdańsk, Poland; ^2^ Department of Psychiatry, University of Oxford, Oxford, United Kingdom; ^3^ Division of Developmental, Psychotic and Geriatric Psychiatry, Department of Psychiatry, Medical University of Gdańsk, Gdańsk, Poland; ^4^ Independent Practitioner, Gdańsk, Poland; ^5^ Department of Nuclear Medicine, Medical University of Gdańsk, Gdańsk, Poland; ^6^ Adult Psychiatry Scientific Circle, Division of Developmental, Psychotic and Geriatric Psychiatry, Department of Psychiatry, Medical University of Gdańsk, Gdańsk, Poland; ^7^ Department of Internal and Paediatric Nursing, Faculty of Health Sciences, Medical University of Gdańsk, Gdańsk, Poland; ^8^ Dialysis Centre Fresenius Ostróda, Ostróda, Poland

**Keywords:** depression, screening, haemodialysis, compliance, dementia, BDI-II, MADRS

## Abstract

**Background:**

Depressive disorder is common among haemodialysis patients. The purpose of this study was to explore approaches to diagnosing depression in the context of a real-life setting, with the view of creating practical recommendations. It also aimed to evaluate the prevalence of depression and dementia.

**Methods:**

We conducted a cross-sectional study in two Dialysis Centres in Poland. Cognitive functions were evaluated using Mini–Mental State Examination (MMSE). The screening for depressive symptoms was assessed using Beck Depression Inventory II (BDI-II). The diagnosis of major depressive disorder was confirmed by a psychiatrist using Diagnostic and Statistical Manual of Mental Disorders 5 (DSM-5). Sociodemographic and clinical data were also collected.

**Results:**

Initially, 136 patients agreed to participate in the study. Dementia was found in 13% of the study group. Sixty-two patients did not agree to perform all the proposed tests and were not included in the analysis, which eventually consisted of 70 patients. According to BDI-II, depressive symptoms were present in 35.7% of patients, while the diagnosis of major depressive disorder (MDD) was confirmed by the psychiatrist in 25.7%. According to the ROC analysis the optimal cut-off score for diagnosing MDD using BDI-II was ≥13 points.

**Conclusions:**

This study suggests that the regular screening for depressive symptoms, followed by a psychiatric consultation in selected patients, might improve diagnosing depression with the goal of achieving a higher quality of life and a lower mortality rate. It may also be a cost-effective model for the management of depression among the haemodialysis population.

## Introduction

Major depressive disorder (MDD) is a common psychiatric condition, with a twelve-month prevalence of around 5–10% and lifetime prevalence of around 20% (1–3). Among outpatients with general medical disorders the prevalence of depression largely exceeds that in the general population, reaching above 50% in groups with certain conditions (4). Given the burden of symptoms, high numbers of sufferers, and the fact that depression often goes undetected, the development of effective strategies for early identification and management of depression in such populations has become an important goal of clinical research (4).

Kidney diseases are among somatic disorders associated with high levels of depression, in particular in their more advanced chronic stages. Chronic kidney disease (CKD) has become a growing public health problem, with the number of individuals with all-stage CKD reaching almost 700 million and over 3 million patients requiring dialysis in 2017 (5). This number is expected to keep rising over the next decade to reach over 5.4 million by 2030, driven by the aging of population and increasing incidence of diabetes and hypertension (6). Among users of in-centre maintenance haemodialysis (MHD) - the most common form of renal replacement therapy (RRT) (7, 8)-depression is present in up to 40% of patients (9).

Estimates of depression prevalence among MHD users however vary between studies. One possible explanation is that depression may be not accurately diagnosed. This may be related to the presence of overlapping somatic symptoms, or differences in the diagnostic processes, such as assessment techniques, diagnostic tools, definitions and thresholds used (10, 11). It has been shown that the use of self- or clinician-administered questionnaires leads to higher estimations of depression compared with the interview-based diagnosis using Diagnostic and Statistical Manual of Mental Disorders 5 (DMS-5) criteria (12). Inaccurate diagnosing of depression has important clinical implications. Particularly noteworthy is the strong correlation between depression and all-cause mortality risk in patients receiving MHD (13, 14), with affective and cognitive symptoms of depression being a better predictor of long-term mortality than its somatic symptoms (15). Depressive symptoms are also independently associated with dialysis nonadherence, lower health resource utilisation (16) and decreased quality of life (17). Performing routine screening for depression has been recommended by Centres for Medicare and Medicaid Services in the United States (18) and in Kidney Disease Outcomes Quality Initiative (KDOQI) guidelines for cardiovascular disease in dialysis patients (19). Nevertheless, depression screening and integrated management of depression in the dialysis population remain insufficient (20, 21).

In the dialysis centres, where access to psychiatric care is often limited, diagnostic scales are frequently used to screen for depression in end-stage renal disease (ESRD). A number of screening tools have been used, including the Cognitive Depression Index (CDI) (22, 23),, the Center for Epidemiological Studies Depression Scale (CES-D) (24), the Hospital Anxiety and Depression Scale-Depressive Subscale (HADS-D) (25), Geriatric Depression Scale 15 (GDS-15) (26), and Initial Depression Inventory- Maintenance Haemodialysis (ID-MHD) (27). The most commonly used assessment tool has however been the self-administered Beck Depression Inventory, both in its original (BDI) and revised (BDI-second edition, BDI-II) versions (11, 28–31). Only few studies compared the consistency of diagnostic outcomes between different scales and clinical diagnosis made by a psychiatrist using DSM criteria (22, 23, 32, 33). Most of the studies performed in the elderly MHD population (26) suggested that in order for BDI and BDI-II to be valid tools comparable to the clinical interview, a traditional cut-off score of 14 points needed to be replaced by the threshold of 15 points or higher (22, 32, 33). This is mostly related to the impact of depressive somatic symptoms common in the MHD population. Establishing a threshold is crucial for promoting the use of BDI-II as a reliable screening tool. This should have important clinical value as on-dialysis assessments using BDI-II, thanks to the ease of using the scale, could constitute a convenient screenings procedure encouraging regular evaluation during the dialysis session, at a time when patients are easily accessible (22). There are some considerations when assessing patients for depression, especially in the elderly population. An important one is the presence of dementia. Approximately thirty percent of haemodialysis patients suffer from dementia (34, 35), with even higher rates among older patients and those with severe somatic conditions (36). The limitations of the use of self-administered rating scales should be considered in this context. Excluding cognitive impairment before making the diagnosis of depression seems to be of significant importance (37). The purpose of this study was to explore commonly used approaches to diagnosing depression in the context of the real-life setting of the haemodialysis centre, with the view of contributing to the development of practical recommendations for depression assessment in haemodialysis patients. To achieve this, we compared recognition of depression using common approaches, specifically the self-rated questionnaire BDI-II, chosen because it remains an easy-to-administer, commonly used tool in the everyday clinical practice, with psychiatrist led assessment based on DSM-5 criteria. Additionally, individuals with and without depression were compared in terms of sociodemographic and clinical features. Dementia screening using Mini-Mental State Examination (MMSE) (38) and clinical evaluation was conducted in order to exclude its impact on depression screening. The study was designed to reflect the daily operating conditions of the haemodialysis centre.

## Materials and methods

### Study population

Adult ESRD patients from two Haemodialysis Centres were enrolled into the study. Individuals over 18 years old, with proper speaking abilities who had been receiving haemodialysis for at least 3 months were recruited consecutively by the medical staff of the dialysis centre. Patients with major psychiatric disorders other than depression, or those with visual or hearing impairments that prevented them performing the tests, were excluded. The study group received the high-flux haemodialysis or hemodiafiltration three times weekly. All participants gave their written informed consent for inclusion. The study was conducted in accordance with the Declaration of Helsinki, and the protocol was approved by the Independent Bioethics Committee for Scientific Research of Medical University of Gdansk.

### Procedure and measures

The study was conducted in two Dialysis Centres in Poland (NZOZ Diaverum Gdańsk and Fresenius Nephrocare Ostróda). All the tests and interviews during the dialysis sessions were performed at least one hour after the initiation and one hour before the termination of the procedure, in order to reduce the risk of intradialysis hypotension impacting cognition. Before performing the depression screening, the MMSE was administered to all the patients by a trained researcher, in order to evaluate their cognitive function. Patients meeting the criteria of moderate or severe dementia (score <19 points) were excluded from the depression evaluation, to avoid the risk of dementia’s impact on their ability to retrospectively assess their mental state. Individuals with mild cognitive impairment (MCI) (24–26 points) and mild dementia (19–23 points) remained in the study, due to the possibility of pseudodementia in the course of MDD. Depression was initially assessed with Beck Depression Inventory II (29). We used a validated version of BDI-II, the language used to collect the data was Polish. The BDI–II is a self-reported, 21-item inventory designed to assess the presence and severity of depressive symptoms. BDI-II assesses symptoms within cognitive, affective and somatic domains. Each item is rated from 0 to 3 points, and the total score ranges from 0 to 63. The score ≥14 points in BDI-II was interpreted as the presence of depressive symptoms. The original authors of the BDI-II recommended the following practical interpretation of their instrument: 0–13 points considered as none to minimal range depression, 14–19 mild depression, 20–28 moderate and 29–63 severe depression (29, 39, 40). All the patients, regardless of the BDI-II score, were subsequently invited for the clinical assessment performed by a senior psychiatrist, experienced in diagnosing MDD and assessing patients with chronic somatic illnesses. The psychiatrist was blind to BDI-II results. The psychiatrists conducted the diagnostic interview based on the DSM-5 criteria (41, 42), and subsequently applied the Montgomery-Asberg Depression Rating Scale (MADRS) to establish the severity of depression (43). MADRS is a standard in monitoring depressive symptoms and has been used in haemodialysis populations in previous studies (44, 45). The psychiatrists additionally used the Clinical Global Impression - Severity scale (CGI-S) to evaluate the severity of depression (46) and the Personal and Social Performance Scale (PSP) (47) to assess psychosocial functioning. The same procedure was followed by all three psychiatrists involved in the study. All the patients diagnosed with depression (regardless of the severity) were offered to remain under psychiatric care.

In one of the centres psychiatrists examined patients within the dialysis setting during the haemodialysis sessions. In the other the psychiatrist was only available in the ambulatory, which meant that patients had to attend a separate session that was scheduled independently of the dialysis treatment. In this centre, none of the patients who filled out the BDI-II questionnaire during their haemodialysis session agreed to the ambulatory clinical evaluation.

The demographic, clinical and laboratory data of the study group were obtained (**Tables 1**, **2**). The severity of comorbidities was scored with the use of Charlson Comorbidity Index (CCI) (48), adjusted for age.

**Table 1:** Clinical data. Comparison between the non-depressed (MADRS < 10 or CGI < 3) and depressed (DSM-5 criteria, MADRS ≥ 10 and CGI ≥3) patients.

Parameter	Whole group *n*=70	Non-depressed *n=*52	Depressed *n=*18	p-value
BMI	26.9 (4.9)	27.3 (4.9)	25.8 (4.8)	0.294813^t^
Diabetes n, (%)				0.485198^c^
no yes	38 (54.2%) 32 (45.7%)	30 (57.7%) 22 (42.3%)	8 (44.4%) 10 (55.6%)	
CCI 1-2 mild 3-4 moderate >= 5 severe	6 (5; 8) 4 (5.7%) 9 (12.9%) 57 (81.5%)	6 (5; 8) 3 (5.8%) 6 (11.5%) 43 (82.7%)	6.5 (5; 8.5) 1 (5.6%) 3 (16.7%) 14 (77.8%)	0.684375^w^
Dialysis duration (years)	3 (2; 4.8)	3 (2; 4)	3.6 (2.2)	0.489249^w^
ESRD cause n, (%)				0.097571^f^
Diabetes GN Hypertension PKD Ischemic nephropathy Nephrolithiasis Reflux nephropathy Interstitial nephritis Other Unknown	19 (21.1%) 17 (24.3%) 4 (5.7%) 5 (7.1%) 5 (7.1%) 2 (2.8%) 3 (4.3%) 4 (5.7%) 6 (8.6%) 5 (7.1%)	12 (23.1%) 12 (23.1%) 3 (5.8%) 3 (5.8%) 4 (7.7%) 1 (1.9%) 3 (5.8%) 4 (7.7%) 6 (11.5%) 4 (7.7%)	7 (38.9%) 5 (27.8%) 1 (5.5%) 2 (11.1%) 1 (5.5%) 1 (5.5%) 0 (0.0%) 0 (0.0%) 0 (0.0%) 1 (5.5%)	
Vascular access n, (%)				0.58603^f^
Arteriovenous fistula	25 (35.7%)	17 (32.7%)	8 (44.4%)	
Permanent catheter Temporary catheter	44 (62.9%) 1 (1.4%)	34 (65.4%) 1 (1.9%)	10 (55.6%) 0 (0.0%)	
Hb g/dl N: F 11.5-16.5, M 13.0-18.0	10.8 (1.3)	10.9 (1.4)	10.5 (1)	0.164954^t^
WBC G/l N: 4.0-11.0	6.8 (5.8; 8.1)	6.7 (5.8; 7.9)	7.3 (5.1; 8.2)	0.869732^w^
Albumin g/l N: 35-52	39.9 (3.8)	39.9 (3.6)	39.6 (4.4)	0.806852^t^
CaxPi	46.6 (13)	46.8 (11.7)	46.1 (16.4)	0.857303^t^
Kt/V >1,2	1.5 (1.2; 1.7)	1.5 (0.3)	1.5 (1.2; 1.7)	0.967294^w^
MMSE	28 (26; 29)	28 (25.5; 29)	27.5 (26.8; 29)	0.961678^w^
BDI-II	10 (7; 16.5)	10 (6; 14)	14.2 (8.2)	0.208242^w^
PSP	60 (46; 84)	67.5 (50; 86.2)	55 (45; 60.2)	0.013187^w*^

Samples from normally distributed populations are described using the mean and standard deviation. The remaining values of quantitative variables were described with the median and the first and third quartiles. n - the number of patients in the group, c - Pearson’s χ2 test with Yates’ continuity correction, w - Wilcoxon rank sum test with continuity correction, t - Student’s t-Test, f - Fisher’s exact test for count data, BMI- body mass index, CCI- Charlson Comorbidity Index, ESRD- end-stage renal disease, PKD- polycystic kidney disease, Hb- hemoglobin, N-normal range, CaxPi-calcium-phosphate index, Kt/V- dialysis adequacy, MMSE- Mini-Mental State Examination, BDI-II-Beck Depression Inventory II, PSP- Personal and Social Performance Scale

*- statistically significant

**Table 2:** Sociodemographic data. Comparison between non-depressed (MADRS < 10 or CGI < 3) and depressed (DSM-5 criteria, MADRS ≥ 10 and CGI ≥3) individuals.

Parameter	Non-depressed	Depressed	p-value	
Gender (n=70)			0.733087^c^	
female	16 (30.8%)	7 (38.9%)		
male	36 (69.2)	11 (61.1%)
Age (years) (n=70)			0.128411^w^	
Smoking (n=57)	70 (62.8; 76)	62.7 (12.5)	0.441555^f^	
no	34 (77.3%)	8 (61.5%)		
yes	6 (13.6%)	3 (23.1%)		
in the past	4 (9.1%)	2 (15.4%)		
Living (n=53)			1^f^	
alone	6 (16.7%)	3 (17.6%)		
with relatives	30 (83.3%)	14 (82.4%)		
Martial status (n=58)			0.257376^f^	
single	5 (11.9%)	3 (18.8%)		
married	29 (69.0%)	7 (43.8%)		
divorced	2 (4.8%)	2 (12.5%)		
widowed	6 (14.3%)	4 (25.0%)		
Residence (inhabitants) (n=52)			0.682529^f^	
<1.000	5 (13.5%)	1 (6.7%)		
1.000–10.000	5 (13.5%)	1 (6.7%)		
>10.000–100.000	3 (8.1%)	0 (0.0%)		
>100.000	24 (64.9%)	13 (86.7%)		
Work (n=66)			1^f^	
active	7 (14.6%)	2 (11.1%)		610
not active	41 (85.4%)	16 (88.9%)		611
Education (n=64)			0.097571^f^	612
primary school	3 (6.2%)	3 (18.8%)		613
vocational school	15 (31.2%)	3 (18.8%)		614
high school	21 (43.8%)	4 (25.0%)		
higher education (BA,MA,PhD)	9 (18.8%)	5 (31.2%)		615 616
no education	0 (0.0%)	1 (6.2%)		617

Percentages apply to the represented group (non-depressed or depressed); c - Pearson’s χ2 test with Yates’ continuity correction, w - Wilcoxon rank sum test with continuity correction, f - Fisher’s exact test for count data, n – numbers for whom information was available, BA- Bachelor of arts, MA-Master of arts, PhD- Doctor, ESRD- end-stage renal disease, DM- diabetes mellitus, PKD- polycystic kidney disease.

### Statistical analysis

The statistical analysis was performed using the functions and procedures of the R package (49). Differences between both groups of quantitative variables were tested using the Wilcoxon test or Student’s t-test. The appropriate tests (and additional options) were selected depending on the p value of the Shapiro-Wilk test and the homogeneity of the variance test. If the samples came from a normally distributed population, the basic features describing the variables were mean values and standard deviations. In other cases, values are described using the median as well as the first and third quartiles. The Pearson’s χ2 test with Yates’ continuity correction and the Fisher’s exact test for count data were used to test the independence of qualitative variables collected in tables presenting the size of individual groups. The research also used ROC analysis and logistic regression (50). For each of the tests used, the level of significance was set at α = 0.05.

## Results

Initially, 136 patients from two Dialysis Centres agreed to participate in the study. Four patients were diagnosed with moderate or severe dementia and were excluded from further evaluation. Patients diagnosed with MCI (14.2% of the whole group), mild dementia (12.9%), and those who did not agree to the MMSE screening (10%), remained in the study. Sixty-two patients (47%) did not agree to participate in some of the study procedures and were excluded from the analysis. Seventy patients (51.5%) who performed all depression evaluations were included in the final analysis. This group consisted of 23 females (32.8%) and 47 males (67.1%). The median age of participants was 69 years.

The numbers of patients with depression diagnosed using BDI-II only, differed from the numbers diagnosed by the psychiatrist using DSM-5 criteria and the scores of MADRS≥10 and CGI ≥3.

Forty-five patients (64.3% of the whole group) had BDI-II score of 0–13, which corresponded to no depression or minimal symptoms. Out of this group, 9 (12.9% of the whole group) were diagnosed with depression by a psychiatrist using the DSM-5 criteria and the scores of MADRS≥10 and CGI ≥3.

Twenty-five patients (35.7% of the whole group) scored ≥14 points on BDI-II, consistent with the cut-off point for depression diagnosis. Of these patients, nine (12.9% of the whole group) were diagnosed with depression by a psychiatrist based on the DSM-5 criteria and scores of MADRS≥10 and CGI ≥3.

In more detail, 13 patients (18.6% of the whole group) had the BDI-II score of 14–19, which corresponded to mild depression. Of these, 4 (5.7% of the whole group) were diagnosed with depression by a psychiatrist based on the DSM-5 criteria and scores of MADRS≥10 and CGI ≥3.

BDI-II criteria for moderate (20–28) and severe (29–63) depression were met by, respectively, 8 patients (11.4% of the whole group) and 4 patients (5.7% of the whole group). Of these, respectively, 4 (5.7% of the whole group) and 1 (1.4% of the whole group) were diagnosed with depression by a psychiatrist based on the DSM-5 criteria and scores of MADRS≥10 and CGI ≥3.

Subsequently, we analysed the differences in qualitative and quantitative data between groups with and without depression based on the diagnosis made by the psychiatrist (depression: DSM-5 criteria, MADRS≥10 and CGI ≥3; no depression: remaining patients). The basic characteristics (sociodemographic and clinical data) for the whole group, as well as depressed and non-depressed subgroups, are presented in **Tables 1** and **2**.

In the whole group, the most common known primary cause of ESRD was diabetes mellitus (DM) (45.7% of all participants) and glomerulonephritis, followed by hypertension, policystic kidney disease and ischemia. The dominant vascular access was the permanent catheter. The CCI score interpreted as the severe comorbidity was found in 81.5% of participants, while mild comorbidity was found in in 5.7%.

The only statistically significant difference for quantitative variables was observed for the PSP score, which suggests that functioning of depressed and non-depressed patients differs across the dimensions assessed by the scale (socially useful activities, personal and social relationships, self-care, disturbing and aggressive behaviours). There were no statistically significant differences between depressed and non-depressed groups in terms of other quantitative variables, as well as qualitative variables, as shown in **Tables 1** and **2** (all p-values >0.05). The number of patients with diabetes mellitus did not differ between groups. We also found no statistically significant differences in the albumin level, body mass index or comorbidity. However, in both groups the average CCI score met the criteria for severe comorbidity.

**Figure 1:**
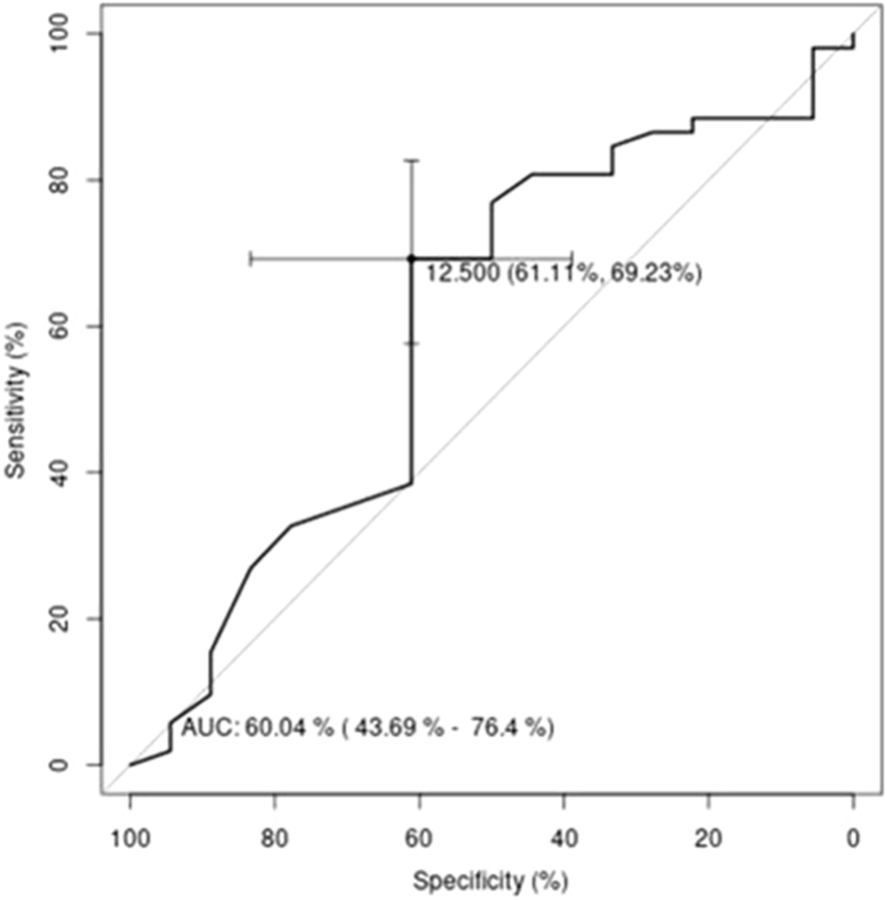
ROC curve for the logistic regression model. The independent variable is the BDI-II score and the dependent variable is membership of the group “no depression” or “depression”.

Using ROC analysis and logistic regression, the possibility of creating a potential tool to determine the absence of depression in dialysis patients based on the BDI-II score variable was examined. For this purpose, a logistic regression model was created, in which the dependent variable was membership of the group (with or without depression), determined using scores on the MADRS and CGI-S scales, as described above. The independent variable was the BDI-II score. Based on the model created in this way, an ROC curve was plotted and the cut-off point for the obtained model was calculated (**Figure 1**).

The cut-off point established for BDI-II was the value dividing patients into two groups. Patients with predicted depression were those with a BDI-II score of 13 or greater and the remaining patients with scores of less than 13 were classified as ‘predicted no depression’. Contingency tables were created on the basis of the groups defined in this way (**Table 3**).

**Table 3:** Contingency table for the Beck variable.

Parameter	No depression (MADRS <10, CGI<3)	Depression (MADRS ≥10, CGI ≥3)	p-value
BDI-II			
nd d	36 16	7 11	0.045667^c^

nd, no depression; d, depression.

The result of the independence test was statistically significant. It indicated that there is a relationship between the groups defined by BDI-II and the groups defined by the MADRS and CGI-S scales. The quality of the classification was assessed by determining values for parameters such as: TPR=69.2% (True Positive Rate - associated with the group “no depression”), TNR=61.1% (True Negative Rate - associated with the group “depression”), PPV=83.7% (Positive Predictive Value) and NPV=40.7% (Negative Predictive Value). In the created model for grouping the values of the BDI-II score variable, only the parameters assessing the classification into the “no depression” group could be considered satisfactory.

## Discussion

In the studied population of patients with ESRD undergoing MHD, 35.7% had BDI-II scores corresponding to mild depression (the cut-off point of 14 points), with 17.1% with scores indicating moderate or severe depression (≥20 points). The number of patients diagnosed with MDD by the psychiatrist using DSM-5 criteria and scores of MADRS≥10 and CGI ≥3 however differed from this, with 25.8% diagnosed with depression. With the exception of PSP score, no statistically significant differences were found in terms of the sociodemographic and clinical features between the depressed and non-depressed patients. In our study, using ROC analysis and logistic regression, the suggested optimal cut-off point for BDI-II, which indicated patients without depression in the clinical evaluation, was 13 points. The compliance rate was low; 47% of the patients, that had agreed to participate in the study, refused to perform some of the proposed tests. MCI and mild dementia were observed in 27.1% of patients.

We used BDI-II as the screening tool as despite its limitations (11, 27), it remains a commonly used measure to explore depressive symptoms in the clinical settings, and this study was designed to reflect the common practices. Using the MADRS scale alone, applied by the psychiatrist, the percentage of individuals with depression reached 35.7%, equal to the number identified in BDI-II screening. These results are comparable with the prevalence of depressive symptoms observed in outpatient populations across different specialties [4]. Psychiatric evaluation attributed diagnosis of MDD based on DSM-5 criteria, with MADRS and CGI scores to assess severity, to 25.8% patients, hence lower than when the scales were used alone. This finding is consistent with previous reports of higher depression rates when self-reported questionnaires were used, compared to the clinical evaluation (12). Such discrepancy may be linked to a number of factors, specific to the researched population. Relevant to our studied population, somatic symptoms may get reported through questionnaires, while in fact they are related to the somatic condition, rather than depression. Indeed, numerous somatic comorbidities were seen in our tested population, and the CCI score indicated severe comorbidity in 81.5% of patients, consistent with the epidemiological data (7). Although the groups with and without depression did not differ in terms of clinical features, people with depression may be more sensitive to somatic problems (for example, it was shown that depression increases sensitivity to pain) (51), which may translate to allocating higher scores to somatic items, whereas a trained psychiatrist may have more clarity whether reported symptoms are related to depression or not. We noted that the number of patients meeting the criteria for depression according to MADRS alone, was equal to the number of patients whose scores on BDI-II indicated depression (35.7% in both cases). At the same time, only 27.8% of patients were diagnosed with depression by the psychiatrist using DSM-5 criteria. This suggests no superiority of clinician applied scales as compared with self-rated scales, and indicates that a full clinical assessment is warranted for a diagnosis. Importantly, this also suggests that some haemodialysis patients whose scores on depression assessment scales, whether self-rated or clinician applied, were indicative of depression, might not in fact have MDD. In such cases, starting depression treatment may be ineffective or even harmful, with the symptoms presented possibly reflecting mild depression, requiring psychological therapy rather than pharmacotherapy. Patients may also be suffering from dementia or yet another mental disorder requiring a different therapeutic approach altogether. The lack of precise diagnostics in the studies on the efficacy of antidepressant treatment in this group of patients may be one of the reasons for the ineffectiveness of therapy, giving false negative results (52).

An intriguing observation was that while BDI-II suggested depression (score ≥14) in a higher number of patients than later confirmed by a psychiatrist, some patients who had scores on BDI-II corresponding to no or minimal depression (≤13), were diagnosed with depression in a psychiatric assessment. This suggests that a psychiatrist may recognise depression based on DSM-5 criteria, when the key symptoms are present, which however may not reach the severity required for the diagnosis using BDI-II. Whether diagnosing patients with such low severity of symptoms is important, for example in the context of offering counselling, is an important questions, however beyond the scope of this study.

Unfortunately, in reality, availability of psychiatric support is low and regular psychiatric assessments in physical health settings are rare. Using a self-rated scale such as BDI-II would help identify patients who could potentially benefit most from a psychiatric assessment, hence focusing the resources available. Therefore, the regular screening using self-administered questionnaires is advisable. BDI-II is easy to perform and can be done while patients are having their haemodialysis session, with no additional burden on their time. The value of performing such a screening cannot be overappreciated. Depression has many adverse effects, from its intrinsic impact on well-being to the worsening of physical health and increased non-compliance with treatments. Therefore its recognition and management are crucial for regular clinical practice and the management of somatic health problems.

According to our results, the optimal cut-off point for BDI-II in diagnosing MDD in the haemodialysis population was equal to or greater than 13 points. It is similar to the one recommended in the general population (i.e. 14 points) (29, 39) and lower compared with cut-off points suggested in the previous studies (22, 32, 33).

Our studied group of patients had high average age and high rate of severe comorbidities. Population aging and increases in comorbidities, observed in patients receiving renal replacement therapy, is in line with changes in the general population. Both have been identified as risk factors for developing depression (7, 53, 54). In both groups the average CCI score suggested severe comorbidity, although with no statistical differences between depressed and non-depressed groups. One such comorbidity, present in the high percentage of patients in our group, was diabetes mellitus. DM is the most common cause of ESRD. Although our study did not identify statistical differences in any of the clinical factors between depressed and non-depressed groups, some of the aspects of somatic health warrant attention. For example, cardiovascular problems are a risk factor for depression, hence an assessment of their indices, especially modifiable ones, is potentially important (55). Phosphate retention is a risk factor for cardiovascular mortality in patients with CKD, and can be potentially modified with diet (56, 57). Although this study did not allow for an assessment of the relationship between adherence to dietary recommendations, which influences phosphate levels, and the occurrence of depressive symptoms, this might be an important practical aspect of future studies. There were no significant between-group differences in other parameters suggested to be associated with future cardiovascular events, such as albumin and haemoglobin levels (57). We have not found statistically significant differences between depressed and non-depressed patients according to Kt/V, used to evaluate haemodialysis adequacy. However, its interpretation as a single parameter has many limitations (58).

One of the reasons for the lack of identification of between-group differences might be the small study sample. Almost half of the recruited patients were lost to the analysis as they refused to perform one or more of the required tests. Our observations of low compliance rate regarding diagnosing depression are consistent with the previous research (59, 60). In our studied population, almost half of the patients who initially gave their written consent to participate, did not agree to follow through with the further psychiatric evaluation. This seemed to be associated with the setup of the assessment. While 84% of patients agreed to a psychiatric assessment provided during their dialysis session, none of the patients followed up with a psychiatric assessment if it was offered in the ambulatory. One possible explanation may be the reluctance to use additional services, considering the time that patients already devote to the regular dialysis procedure. The attitudes to psychiatric assessments may also play a role. Interestingly, none of the patients from the dialysis centre which was in a small town – and incidentally where the assessment was offered in the ambulatory only – agreed to see the psychiatrist. Although not examined in the study, the stigma around psychiatric assessments is still an important issue, and may be more pronounced in particular social contexts. Even if the examination during a dialysis session is challenging given the lack of privacy in the setting where a number of patients share the dialysis room, as well as the noise of regular hospital activities, it seemed to be more acceptable for the patients in our study than when a separate appointment was required.

Therefore, on-dialysis consultations may be recommendable (22).

Our study included dementia screening using MMSE. The percentage of patients diagnosed with MCI or mild dementia was 27.1%. An assessment of cognitive impairments when assessing depression, especially in the elderly populations, is important, as dementia can distort the diagnosis of depression and influence the results of its treatment. At the same time, the results of MMSE have to be considered carefully as higher scores may be related to pseudo-dementia in the course of depression, as well as be related to the conditions of the examination itself. Previous research showed that dialysis patients with greater burdens of depressive symptoms performed worse in cognitive tests related to processing speed and executive function (61). Therefore, individuals who met the criteria of MCI and mild dementia in MMSE were not excluded.

In the real world, the access to a psychiatric assessment in the dialysis setting is very restricted. Employing a psychiatrist full-time is usually not considered cost-effective; examining all the patients would be associated with high costs and an additional pressure on scarce resources. However, given how common depression is in this population of patients, and how big its impact on general health is, an identification of depressed individuals is an important issue. One solution, supported by this study, could be the regular screening for depression using self-reported scales, applied during the dialysis session, in order to identify patients requiring a psychiatric consultation. Our results suggest that this consultation might be best offered during the dialysis session, as shown by differences in accepting an assessment depending on the setting during and outside of the dialysis session. Such models could be more beneficial both for patients and the service providers. Increasing the availability of psychiatric consultations in the dialysis settings for the selected patients, might improve the effectiveness of diagnosing and treating depression in this population, and in turn have an impact on physical health and treatment adherence. The proper screening algorithm might improve the treatment of depression and have an impact on patients’ quality of life as well as compliance with the dialysis recommendations.

One of the study limitations was the small sample size, discussed above. The others were related to the differences between the two settings regarding the place and time of the psychiatric evaluation, which may be the reason why patients from one of the settings refused a psychiatric consultation. Performing the clinical evaluation during the dialysis session might be considered a limitation as well, given a relative lack of privacy and other factors discussed above. However, as our study suggested, in real life, even if such conditions are not ideal, this approach may still be more acceptable for patients than a separate appointment.

The future research on depression screening in the haemodialysis patients leading to the development of the practical recommendations regarding diagnosis and treatment, is an important clinical need. It might also be of importance to explore the correlation between depression and the compliance to the dialysis treatment itself.

## Conclusions

This study suggests that, although the psychiatric assessment is considered superior to using depression rating scales, the regular use of self-rated questionnaires to assess depression during haemodialysis sessions may help to identify a subgroup of patients with suspected depression, who would benefit from a psychiatric assessment. This might be a cost-effective model for the management of depression among the haemodialysis population. Performing dementia screening should be taken into consideration before diagnosing depressive disorders. The regular screening for depressive symptoms, followed by a psychiatric consultation in selected patients as a part of regular care in Dialysis Centre, might improve diagnosing depression, with the goal of achieving a higher quality of life, better treatment adherence, and a lower mortality rate.

## Data availability statement

The original contributions presented in the study are included in the article/supplementary material. Further inquiries can be directed to the corresponding author.

## Ethics statement

The studies involving humans were approved by Independent Bioethics Committee for Scientific Research of Medical University of Gdansk. The studies were conducted in accordance with the local legislation and institutional requirements. The participants provided their written informed consent to participate in this study. Written informed consent was obtained from the individual(s) for the publication of any potentially identifiable images or data included in this article.

## Author contributions

AK: Conceptualization, Data curation, Formal analysis, Investigation, Methodology, Writing – original draft, Writing – review & editing. MR: Formal analysis, Funding acquisition, Supervision, Writing – review & editing. BG: Data curation, Formal analysis, Methodology, Supervision, Validation, Writing – review & editing. PP: Data curation, Formal analysis, Investigation, Writing – review & editing. MP: Conceptualization, Data curation, Investigation, Writing – review & editing. ASK: Data curation, Investigation, Writing – original draft. PW: Data curation, Formal analysis, Methodology, Supervision, Writing – original draft, Writing – review & editing. MB: Data curation, Investigation, Writing – original draft. AM-S: Data curation, Investigation, Writing – original draft. PR: Data curation, Investigation, Supervision, Writing – review & editing. KC: Data curation, Investigation, Writing – review & editing. KB: Data curation, Investigation, Writing – review & editing. JG: Data curation, Formal Analysis, Investigation, Methodology, Supervision, Writing – review & editing.
